# Diagnostic biases in translational bioinformatics

**DOI:** 10.1186/s12920-015-0116-y

**Published:** 2015-08-01

**Authors:** Henry Han

**Affiliations:** Department of Computer and Information Science, Fordham University, New York, 10023 NY USA; Quantitative Proteomics Center, Columbia University, New York, NY USA

**Keywords:** Translational bioinformatics, Omics, Diagnostic biases, Machine learning

## Abstract

**Background:**

With the surge of translational medicine and computational omics research, complex disease diagnosis is more and more relying on massive omics data-driven molecular signature detection. However, how to detect and prevent possible diagnostic biases in translational bioinformatics remains an unsolved problem despite its importance in the coming era of personalized medicine.

**Methods:**

In this study, we comprehensively investigate the diagnostic bias problem by analyzing benchmark gene array, protein array, RNA-Seq and miRNA-Seq data under the framework of support vector machines for different model selection methods. We further categorize the diagnostic biases into different types by conducting rigorous kernel matrix analysis and provide effective machine learning methods to conquer the diagnostic biases.

**Results:**

In this study, we comprehensively investigate the diagnostic bias problem by analyzing benchmark gene array, protein array, RNA-Seq and miRNA-Seq data under the framework of support vector machines. We have found that the diagnostic biases happen for data with different distributions and SVM with different kernels. Moreover, we identify total three types of diagnostic biases: overfitting bias, label skewness bias, and underfitting bias in SVM diagnostics, and present corresponding reasons through rigorous analysis. Compared with the overfitting and underfitting biases, the label skewness bias is more challenging to detect and conquer because it can be easily confused as a normal diagnostic case from its deceptive accuracy. To tackle this problem, we propose a derivative component analysis based support vector machines to conquer the label skewness bias by achieving the rivaling clinical diagnostic results.

**Conclusions:**

Our studies demonstrate that the diagnostic biases are mainly caused by the three major factors, i.e. kernel selection, signal amplification mechanism in high-throughput profiling, and training data label distribution. Moreover, the proposed DCA-SVM diagnosis provides a generic solution for the label skewness bias overcome due to the powerful feature extraction capability from derivative component analysis. Our work identifies and solves an important but less addressed problem in translational research. It also has a positive impact on machine learning for adding new results to kernel-based learning for omics data.

## Background

With the surge of translational medicine and computational omics research, complex disease diagnosis tends to more and more rely on disease signatures discovered from the sheer enormity of high-throughput omics data [1–4]. Identifying disease molecular signatures from different pathological states not only captures the subtlety between disease subtypes and controls, but also provides disease gene hunting, related pathway query, genome wide association (GAWS) investigations, and following drug target identification [5–7]. The translational technologies in medicine along with the exponential growth of high-throughput data in genomics, transcriptomics, and proteomics are preparing for the coming era of personalized medicine to customize medical decisions and practices to individual patients [6, 8].

Although different state-of-the-art classifiers have been widely employed in such a massive data driven disease diagnostics to enhance diagnostic accuracy, there was almost no investigation on their diagnostic biases that are essential for the success of translational medicine [9, 10]. A diagnostic bias simply refers that a classifier cannot unbiasedly conduct diagnosis for a given input omics data in our context. Instead, it may tend to favor some phenotype or even totally ignore the other, even if the diagnostic accuracy appears to be reasonable sometimes.

In other words, given a training data consisting of *m* normalized omics samples *x*_*i*_ and its corresponding labels *y*_*i*_ ∈ {−1,+1}, i.e. $\{x_{i},y_{i}\}{}_{i=1}^{m},$ the decision function *f*(*x*|*x*_1_,*x*_2_⋯*x*_*m*_) inferred from the classifier demonstrates some bias in determining the class type (phenotype) of a new sample *x*^∗^, which is assumed to follow a same normalization procedure as the training data, due to inappropriate parameter choice, model selection, biased label distribution, or even some special characteristics of input data. It is noted that we generally assume all training and testing samples are chosen from a normalized population data for the convenience of diagnosis in our context, which avoids possible renormalization and classifier retraining overhead for the following diagnosis. For example, a diagnostic results: *f*(*x*^∗^|*x*_1_,*x*_2_⋯*x*_*m*_)=1 is probably obtained because almost all training samples are labeled with ^′^+1^′^, even if the true label of *x*^∗^ is *y*^∗^=−1.

As a result, inaccurate or even deceptive diagnostic results would be produced and lead to an inaccurate or even totally wrong clinical decision making. In particular, such a diagnostic bias can happen to any classifiers due to different decision models, input data distributions, and/or model selection choices.

As such, a comprehensive and rigorous investigation on the diagnostic bias problem are an urgent demand from translational research. This is because a robust disease diagnostic requires a classifier achieves both efficiency and security. The efficiency means the classifier can attain a high-level diagnostic accuracy with a good generalization capability. The security refers to the classifier can unbiasedly recognize each label type by avoiding possible biases in the classifier ’s decision function inference. There are quite a lot previous studies done on the efficiency problem, but almost no previous literature addressed the security issue, i.e. the diagnostic bias problem in translational research. In particular, we need to answer the following diagnostic bias related queries: *when will it happen, why does it happen, and how to conquer it and achieve efficiency?*

To answer these key questions, we employ support vector machines (SVM) as a representative in this study to investigate disease diagnostic bias for its rigorous decision model, good scalability, and popularity in translational medicine [11–13]. We present the following novel findings from using benchmark gene array, protein array, RNA-Seq and miRNA-Seq data in this work.

First, diagnostic biases can happen for an SVM classifier under any kernels in different model selections, whereas it is more likely to occur under nonlinear kernels. Given input data with two different phenotypes, diagnostic biases usually reflect as extremely imbalanced sensitivity and specificity values, even if they appear to achieve a reasonable diagnostic accuracy. Moreover, it seems that diagnostic biases are irrespective of data distributions: we have observed it happens to normally distributed and negative binomial distributed data.

Second, there are three types of diagnostic biases: *overfitting bias*, *label skewness bias*, and *underfitting bias* in SVM diagnostics. The overfitting and label skewness biases both demonstrate a majority-count phenotype favor mechanism, i.e., only majority-count samples can be recognized in diagnosis. They are mainly caused by a built-in molecular signal amplification mechanism in omics data profiling, data label skewness, and inappropriate kernel selection respectively.

The built-in signal amplification mechanism is mainly responsible for the overfitting biases. It refers that all high-throughput omics profiling systems employ real-time PCR or similar approaches to amplify gene or protein expression levels exponentially [14, 15]. The data label skewness, which is mainly responsible for the label skewness biases, means that class label distributions are skewed to some specific type of samples (e.g., positive). We define the type of samples with more counts in the label set as the majority-count type for the convenience of description. The inappropriate kernel selection simply means a wrong kernel selection lets the corresponding SVM classifier lose diagnostic capability and result in the underfitting biases.

Third, the label skewness bias is more challenging to detect and conquer because it can be easily confused as a normal diagnostic case from its deceptive accuracy. To tackle this problem, we propose a derivative component analysis based support vector machines (DCA-SVM) to conquer the label skewness bias by comparing its performance with those of the state-of-the-art peers. The proposed DCA-SVM diagnosis not only conquers the label skewness bias but also achieves rivaling clinical diagnostic results by leverage the powerful feature extraction capabilities of derivative component analysis [16].

It is noted that our studies comprehensively identify different diagnostic biases and present novel effective solutions for the important but less addressed problem, Compared with our previous work in conquering SVM overfitting [10], this study provides more systematic and novel results to kernel-based learning for omics data and translational bioinformatics. In particular, our studies firstly identify the label skewness bias that is usually confused as a normal diagnostic case in the past literature and provides a rivaling clinical bias overcome method. As such, it will have positive impacts on translational research and machine learning fields.

## Methods

As a widely used diagnostic method for its good scalability, support vector machines (SVM) can be described as follows. Given a training data set $\{(x_{i},y_{i}\})_{i=1}^{m}$, *x*_*i*_∈ℜ^*n*^ with labels *y*_*i*_∈{−1,+1}, an SVM computes an optimal separating hyperplane: (*w*·*x*)+*b*=0 to attain the maximum margin between the positive and negative observations (samples), where *w* is the normal and bias vector of the hyperplane respectively. The margin refers to the maximal width of two boundary hyperplanes parallel to the optimal separating hyperplane.

If the training data are linearly separable, it is equivalent to finding *w* and *b* that minimize the quadratic programming (QP) problem $\arg \min _{w,b}\frac {1}{2}||w||^{2}$ under the condition $y_{i}(w\cdot x_{i}+b)-1\geqslant 0,$ for each observation *x*_*i*_ in the training data [13]. The QP problem can be solved by seeking solutions of Lagrange multipliers *α*_*i*_≥0,*i*=1,2⋯*m*, in the following dual problem,

(1) $$ \text{max}L_{d}(\alpha)=\sum_{i=1}^{m}\alpha_{i}-\frac{1}{2}\sum_{i=1}^{m}\sum_{j=1}^{m}\alpha_{i}\alpha_{j}y_{i}y_{j}(x_{i}\cdot x_{j})  $$

where *w* and *b* can be calculated by $w=\sum _{i=1}^{m}\alpha _{i}y_{i}x_{i}$ and *y*_*i*_(*w*·*x*_*i*_+*b*)−1=0 respectively. As a result, the class type of an unknown sample *x*^′^ can be determined as $f(x')=sign((\sum _{i=1}^{m}\alpha _{i}(x_{i}\cdot x')+b).$ That is, the support vectors, which are the training samples *x*_*i*_ corresponding to *α*_*i*_>0, totally determine diagnostics according to the spatial locations of test samples with respect to them. Geometrically, the support vectors are the data points that are closest to the optimal separating hyperplane and can be usually identified in corresponding visualization.

If the training data are not linearly separable, it means the SVM classifier can find only the optimal separating hyperplane that separates many but not all training samples. In other words, the SVM classifier permits misclassification errors in this soft margin case [12]. Mathematically, it is equivalent to adding slack variables *ξ*_*i*_ and a penalty parameter *C* to the original problem under *L*_1_ or *L*_2_ norms. The penalty parameter *C*, also called the box constraint parameter, is the upper bound of all Lagrange multipliers *α*_*i*_ in the corresponding dual problems.

For example, the original problem is updated as $\arg \min _{w,b,\xi _{i}} \left (\frac {1}{2}||w||^{2}+C\sum _{i=1}^{m}\xi _{i}\right)$ under the conditions $y_{i}(w\cdot x_{i}+b)-1\geqslant \xi _{i}$, and *ξ*_*i*_≥0 under the *L*_1_ norm regularization. Similarly, the original problem is updated as $\arg \min _{w,b,\xi _{i}}(\frac {1}{2}||w||^{2}+C\sum _{i=1}^{m}{\xi _{i}^{2}})$ under the same conditions for the *L*_2_ norm regularization. The *w*,*b* and corresponding support vectors can be obtained by solving its corresponding dual problems [12].

If the training data do not have a simple hyperplane as an effective separating criterion, they can be mapped to a higher or even infinitely dimensional feature space г using a mapping function *ϕ*:*x*_*i*_→г, and constructing an optimal nonlinear decision boundary in г to achieve more separation capabilities. Correspondingly, the decision function for an unknown sample *x*^′^ is formulated as $f(x')=sign\left (\left (\sum _{i=1}^{m}\alpha _{i}(\phi (x_{i})\cdot \phi (x')\right)+b\right).$ Note that the inner product (*ϕ*(*x*_*i*_)·*ϕ*(*x*_*j*_)) in г can be evaluated by any kernel (*ϕ*(*x*_*i*_)·*ϕ*(*x*_*j*_))=*k*(*x*_*i*_,*x*_*j*_) implicitly in the input space ℜ^*n*^ if its corresponding kernel matrix is positive definite, that is, $f(x')=sign\left (\left (\sum _{i=1}^{m}\alpha _{i}k(x',x_{i})+b\right.\right).$

### Kernel selection

Although there are a class of kernel functions available, we mainly focus on the following kernels: a Gaussian radial basis function (*‘rbf*’) kernel: *k*(*x*,*x*^′^)= exp(||*x*−*x*^′^||^2^/2*σ*^2^), quadratic kernel (*‘quad’*) : *k*(*x*,*x*^′^)=(1+(*x*_*i*_·*x*^′^))^2^, multilayer perceptron kernel (*‘mlp’*): *k*(*x*,*x*^′^)= tanh((*x*_*i*_·*x*^′^)−1) kernel, and a widely-used linear kernel: *k*(*x*,*x*^′^)=(*x*_*i*_·*x*^′^), in our experiment. In addition, we design an adjusted Gaussian kernel function: *‘rbf2’*, which is obtained by tuning the bandwidth parameter as the total variations of all *m* training samples: $\sigma ^{2}=\frac {1}{(m-1)^{2}}\sum _{i,j}||x_{i}-x_{j}||^{2} $ in the original Gaussian kernel, to demonstrate the impact of parameter tuning in enhancing SVM diagnosis under the Gaussian *‘rbf’* kernel.

In practice, there are different SVM variants applied in disease diagnosis for its advantages in modeling or implementation. Least-Sequare SVM (LS-SVM) is one of those methods [12, 17]. It only employs equality constraints to reformulate the standard SVM (C-SVM). As a result, the normal *w* and bias *b* of the optimal separating hyperplane are calculated by solving linear systems instead of a quadratic programming problem [18].

Previous results have reported that LS-SVM is comparable to the classic SVM in terms of performance and generalization [12, 18]. In this work, we employ LS-SVM to substitute the classic SVM in disease diagnosis for its efficiency and simplicity [17, 18]. The detailed LS-SVM implementations are chosen from Matlab R2012b bioinformatics Toolbox, which implements the *L*_2_ soft-margin SVM classifier [19].

### SVM classifier parameterization

Since we aim at addressing generic diagnostic biases problems in translational bioinformatics through support vector machines, we do not tend to employ an SVM model with too many parameters or seek very special values in parameter setting to prevent the loss of generalization of results. As such, we employ the LS-SVM model for its built-in advantage in simplifying parameter setting than the other models [17]. Moreover, we choose to set the default parameters generically in the SVM diagnosis to guarantee the reproducibility and generalization of our results.

The most important parameter in our context will be the penalty parameter *C*, which affects the training errors and generalization somewhat directly. A large *C* may produce better diagnostic results but risk the loss of the generalization of the classifier; A small *C* may lead to low diagnostic results but enhance the classifier’s generalization. In our context, the penalty parameter *C* is chosen as 1.0 uniformly in all diagnoses instead of rescaled values for different groups of samples to guarantee comparable results for different data sets that have skewed or balanced label distributions. In particular, such a parameter choice will contribute to more comparable and easily interpretable Lagrange multipliers *α*_*i*_ values that are weights of the support vectors. Although a grid-search way can be employed to seek ‘optimal’ *C* parameters by trying a geometric sequence such as 2^−10^,2^−9^,…2^0^,…2^10^ under a specified cross validation for each data set [13], such an approach may not contribute to generalizable diagnostic results and possible prohibitive training time demand.

Furthermore, we choose to automatically scale the training samples to zero mean and unit variance data before training, which is equivalent to corresponding feature scaling [13], to optimize the kernel matrix’s structure for the sake of learning efficiency and the following diagnostic generalization.

#### Model selection

We employ widely-used cross-validation methods for model selection that include *k*-fold cross-validation (*k*-fold CV) and independent training and test set approach for the sake of comprehensive diagnostic bias investigation, in addition to leave-one-out cross validation (LOOCV). The *k*-fold CV randomly partitions the training data to form *k* disjoint subsets with approximately equal size, removes the *i*^*th*^ subset from the training data and employs the the remaining *k* −1 subsets to construct the decision function and infer the class types of the samples in the removed subset. Moreover, in the independent training and test set approach, we randomly select 50 % of input omics data for training and another 50 % for test, and repeat such a process 500 times for each data to fully investigate different diagnostic biases and validate the effectiveness of our proposed bias-conquering algorithm.

#### Data selection and preprocessing

We firstly choose three benchmark omics data sets: *BreastIBC*, *Hepatocellular carcinoma (HCC),* and *Kidney* in our experiment, which are produced by state-of-the-art gene array, protein array and RNA-Seq technologies respectively [20–22]. Table 1 illustrates the detailed information of the three data sets in platforms, sample distributions, and feature numbers, where a feature refers a gene (probe), *m/z* ratio, or transcript in our context.

**Table 1:** Benchmark data

Data	#Feature	#Sample	Technology	Platform
*BreastIBC*	18,995	13 *inflammatory breast cancer (‘IBC’)*+		
		34 *non-inflammatory breast cancer (‘NIBC’)*	Gene array	Affymetrix GeneChip
*HCC*	23,846	78 *Hepatocellular carcinoma*+ *72 normal*	Protein array	MALDI-TOF
*Kidney*	20,531	*68 normal + 475 kidney renal cell carcinormal tumor*	RNA-Seq	IlluminaGA_RNASeq

It is noted that these data are normalized and processed by different methods. For example, robust multiarray average (RMA) method is applied to normalize the *BreastIBC* data and Reads Per Kilobase per Million mapped reads (RPKM) is used to normalize *Kidney* the data [23–25]. The original raw*BreastIBC* data set has been retrieved from the NCBI Gene Expression Omnibus (GEO) series data with accession number GSE5847, which consists of 13 *inflammatory breast cancer (‘IBC’)* and 34 *non-inflammatory breast cancer (‘NIBC’)* stromal cell samples across 22,283 probes [21, 26]. We have further filtered small-variance genes and obtained our *BreastIBC* data set with 18,995 probes. The *Hepatocellular carcinoma (HCC)* data is a mass spectral proteomic data set generated from the MALDI-TOF platform and its detailed normalization process can be found in Ressom *et al.*’s work [20].

It is noted that both *BreastIBC* and *HCC data* are subject to normal distributions, and the *Kidney* data are subject to negative binomial (NB) distributions approximately [25]. In addition, the sample label distributions of these data are also different. The *HCC data* have an almost balanced distribution: 78 *Hepatocellular carcinoma vs 72 normal*samples. But the *BreastIBC and Kidney* data have obviously skewed label distributions, where the majority count samples are much more than the minority count samples (e.g. 13 *‘IBC’ vs* 34 *‘NIBC’* in the *BreastIBC* data; *68 normal vs 475 renal cell carcinormal tumor* samples in the *Kidney* data).

## Results

We introduce the following set of measures for the sake of diagnostic bias investigations: diagnostic accuracy, sensitivity, specificity, positive predictive ratio (PPR), and negative predictive ratio (NPR). The diagnostic accuracy is the ratio of the correctly diagnosed test samples (targets) over total test samples (targets), i.e. $accuracy=\frac {TP+TN}{TP+FP+TN+FN},$ where TP (TN) is the number of positive (negative) samples correctly diagnosed, and FP (FN) is the number of negative (positive) samples incorrectly diagnosed. The sensitivity, specificity, and positive predictive ratio (PPR) are defined as $sensitivity=\frac {TP}{TP+FN},$ and $specificity=\frac {TN}{TN+FP},PPR=\frac {TP}{TP+FP},$ and $NPR=\frac {TN}{TN+FN}$ respectively. It is noted that we use targets and samples interchangeably in this study.

We conduct SVM diagnosis under a 5-fold cross validation for the three data sets under the following kernels: *‘linear’*, *‘quad’*, *‘mlp’*, *‘rbf’*, and *‘rbf2’*, where the bandwidth parameter *σ*^2^ in the *‘rbf’ and ‘rbf2’* kernels are selected as 1 and the total variations of all training samples respectively. It is noted that each sample in the training data is scaled as a zero mean sample with variance 1.0 before building the optimal separation plane in SVM diagnostics. Table 2 illustrates the SVM diagnoses for the three benchmark data sets with five kernels under the 5-fold cross validation. We have the following interesting findings about diagnostic biases.

**Table 2:** SVM diagnosis for benchmark data under 5-fold cross validation

Algorithm	Accuracy ± std (%)	Sensitivity ± std (%)	Specificity ± std (%)	NPR ± std (%)	PPR ± std (%)
		*BreastIBC data*			
*SVM-linear*	74.56 ± 04.52	97.14 ± 06.39	16.67 ± 23.67	NaN	75.70 ± 06.52
*SVM-rbf*	72.56 ± 03.63	100.0 ± 00.00	00.00 ± 00.00	NaN	72.56 ± 03.63
*SVM-quad*	74.56 ± 04.52	97.14 ± 06.39	16.67 ± 23.67	NaN	75.70 ± 06.52
*SVM-rbf2*	72.83 ± 10.92	85.71 ± 14.29	40.00 ± 09.13	63.33 ± 34.16	78.65 ± 05.88
*SVM-mlp*	45.67 ± 18.09	48.10 ± 22.99	40.00 ± 09.13	25.67 ± 14.02	65.33 ± 12.16
		*HCC data*			
*SVM-linear*	94.02 ± 01.43	95.81 ± 03.83	92.42 ± 05.21	96.17 ± 03.50	92.39 ± 05.00
*SVM-rbf*	52.00 ± 00.75	100.0 ± 00.00	00.00 ± 00.00	NaN	52.00 ± 00.75
*SVM-quad*	82.05 ± 10.66	77.00 ± 10.77	87.52 ± 12.21	77.87 ± 10.32	87.38 ± 11.89
*SVM-rbf2*	89.90 ± 04.32	92.86 ± 08.75	87.17 ± 06.48	93.60 ± 07.25	87.33 ± 05.32
*SVM-mlp*	51.87 ± 10.96	46.00 ± 15.80	58.29 ± 10.06	50.43 ± 08.72	53.44 ± 14.68
		*Kidney data*			
*SVM-linear*	90.23 ± 02.35	96.84 ± 03.07	44.07 ± 06.63	71.46 ± 16.90	92.38 ± 00.71
*SVM-rbf*	87.48 ± 00.44	100.0 ± 00.00	00.00 ± 00.00	NaN	87.48 ± 00.44
*SVM-quad*	87.47 ± 01.70	94.47 ± 01.20	17.47 ± 07.89	50.00 ± 21.21	89.21 ± 00.80
*SVM-rbf2*	87.48 ± 00.44	100.0 ± 00.00	00.00 ± 00.00	NaN	87.48 ± 00.44
*SVM-mlp*	53.39 ± 06.79	54.32 ± 07.79	46.92 ± 10.08	13.02 ± 02.95	87.67 ± 02.47

### Three diagnostic biases

The diagnostic biases would take place in an SVM classifier with any kernels, but it is more likely to occur under nonlinear kernels. In fact, they can happen for almost all SVM classifiers under three different scenarios: *overfitting bias, label skewness bias*, and *underfitting bias*. It is worthwhile to point out that the overfitting bias and label skewness bias may demonstrate similar diagnostic results, whereas they are caused by different reasons.

#### Overfitting biases

The overfitting bias demonstrates the majority-count phenotype favor mechanism in diagnosis under the nonlinear kernels like *‘rbf’*. That is, the SVM classifier will always diagnose an unknown sample as the type of the samples with the majority-count in the training data (e.g., *‘NIBC’* type for the *BreastIBC* data). Finally, its diagnostic accuracy will equal or approximate the majority-count ratio of the input data. For example, the SVM with the *‘rbf’* kernel *(SVM-rbf)* has the diagnostic accuracies that approximate or totally equal to their corresponding majority-count ratios for the three data sets : $72.56\,\%\thickapprox \frac {34}{34+13}=72.34\,\%, 52.00\,\%=\frac {78}{78+72},$ and $87.48\,\%=\frac {475}{475+68}$ respectively.

#### Why does NaN appear in diagnostic results?

The question is why the corresponding *NPR* is NaN in diagnostics (Table 2)? The reason is that the classifier can only recognize the majority-count samples that are specified as the positive type target in our experiment. That is, each trial of diagnoses has a zero count for true negative and false negative, i.e. *TN*=0 and *FN*=0, because all negative targets, which are minority-count samples in our experiment, are diagnosed as the positive type. As a result, $NPR=\frac {TN}{TN+FN}$ will be NaN. So are the corresponding sensitivity values always 100 % $\left (\frac {TP}{TP+FN}=\frac {TP}{TP}=1.0\right)$ and the specificity values 0 % ($\frac {TN}{TN+FP}=\frac {0}{FP}=0.0,$ where *FP* is actually totally number of negative samples that appear as the minority-count samples in our diagnostic experiments).

Similarly, the SVM with the *‘rbf2’* kernel also demonstrates similar diagnostic results as before, where *‘rbf2’* is obtained by tuning the bandwidth parameter in the original Gaussian kernel. Although they may show some improvements for the protein array data (*HCC* data), they still demonstrate the major-phenotype favor mechanism for the gene array and RNA-Seq data. Alternatively, it indicates that simply tuning the bandwidth parameter may not be a good way to conquer such an diagnostic bias.

#### Label skewness biases

Unlike the overfitting bias, the label skewness bias demonstrates two different cases. The first is that the SVM classifiers with a linear or nonlinear kernel (e.g., *‘quad’*) demonstrate *an explicit label skewness diagnostic bias* by presenting a diagnostic accuracy close to the majority-count ratio and a pair of unbalanced sensitivity and specificity. For example, Table 1 shows that both *SVM-linear* and *SVM-quad* classifiers achieve a 74.56 % accuracy that is close to the majority-count ratio: 72.34 *%* with an imbalanced sensitivity 97.14 % and specificity 16.67 % respectively for the *BreastIBC* data. This indicates such a model can recognize few negative targets in one or more diagnostic trials in addition to diagnosing all positive targets and most of negative targets to the positive target type, which is the majority-count type specified in our implementations.

The second is that a linear kernel SVM demonstrates *an implicit label skewness diagnostic bias* by presenting a normal diagnostic accuracy but with a pair of imbalanced sensitivity and specificity. For example, the *SVM-linear* classifier achieves 90.23 *%* accuracy with sensitivity 96.84 % and specificity 44.07 %. Such a result indicates there are a large number of false positives than those of false negatives due to the dominance of the positive type in the training data.

It is noted that not all linear kernels would encounter diagnostic bias. Instead, the *SVM-linear* classifier achieves 94.02 *%* accuracy with 95.81 *%* sensitivity and 94.21 *%* specificity for the *Hepatocellular carcinoma* (*HCC*) data with 78 *HCC* and 72 normal samples that have a more balanced label distribution than those of the *BreastIBC* and *Kidney* data.

#### Underfitting biases

The *underfitting bias* refers that an SVM classifier with a nonlinear kernel such as *‘mlp’* leads to an underfitting model in diagnostics. The model itself is inappropriate for disease diagnostics because the high-dimensional feature selection space generated from the kernel function may distort the information conveyed by the original data [12, 27]. As a result, the SVM classifier will have a quite low diagnostic performance due to the underfitting. For example, the *SVM-mlp* classifier has about 50 % level diagnostic accuracy for all the three data sets. That is, the classifier is equivalent to a random classifier that conducts almost ad-hoc diagnosis because of the underfitting bias.

Finally, it is clear that the diagnostic biases seem to be irrespective of data distributions. They happen for the gene and protein array data that are subject to normal distributions and RNA-Seq count data that are subject to negative binomial (NB) distributions in our experiment [25].

### Diagnostic biases under other cross validations

It is worthwhile to point that diagnostic biases can also happen in other cross validations such as independent training and test set approach and leave-one-out cross validation (LOOCV) besides the *k*-fold cross validation. This is because diagnostic biases may occur in each diagnostic trial under a specific kernel due to the built-in characteristics of input data we will mention in the next section. For example, we generate 100 independent training and test sets for the *BreastIBC* data, where each sample has a 50 % likelihood to be selected in the training and test set. The *SVM-rbf* and *SVM-linear* classifiers has the almost same performance as illustrated in Table 2. For example, the former has the average accuracy: 72.70 % ± 6.48 % with sensitivity: 100.00 ± 0.00 % and specificity: 00.00 ± 0.00 %; the latter has the average accuracy: 73.83 % ± 7.02 % with sensitivity: 92.87 % ± 6.58 % and specificity: 25.45 % ± 15.82 %. It is noted that similar results can be also found for this data set under the LOOCV.

#### What are the reasons for diagnostic biases?

The are different reasons for the three different diagnostic biases, though the overfitting bias and label skewness bias may demonstrate similar diagnostic results.

The reason for the overfitting bias is rooted in the large or even huge pairwise distances *d*_*ij*_=||*x*_*i*_−*x*_*j*_]]^1/2^ between omics samples, which implies that the corresponding distances in the feature space under the *’rbf’* kernel *k*(*x*_*i*_,*x*_*j*_)= exp(−||*x*_*i*_−*x*_*j*_||^2^/2) will be a zero or tiny value approximate to zero. As a result, it leads to an identity or approximately identity kernel matrix that causes the SVM classifier to recognize the majority-count type samples only.

**Fig. 1:**
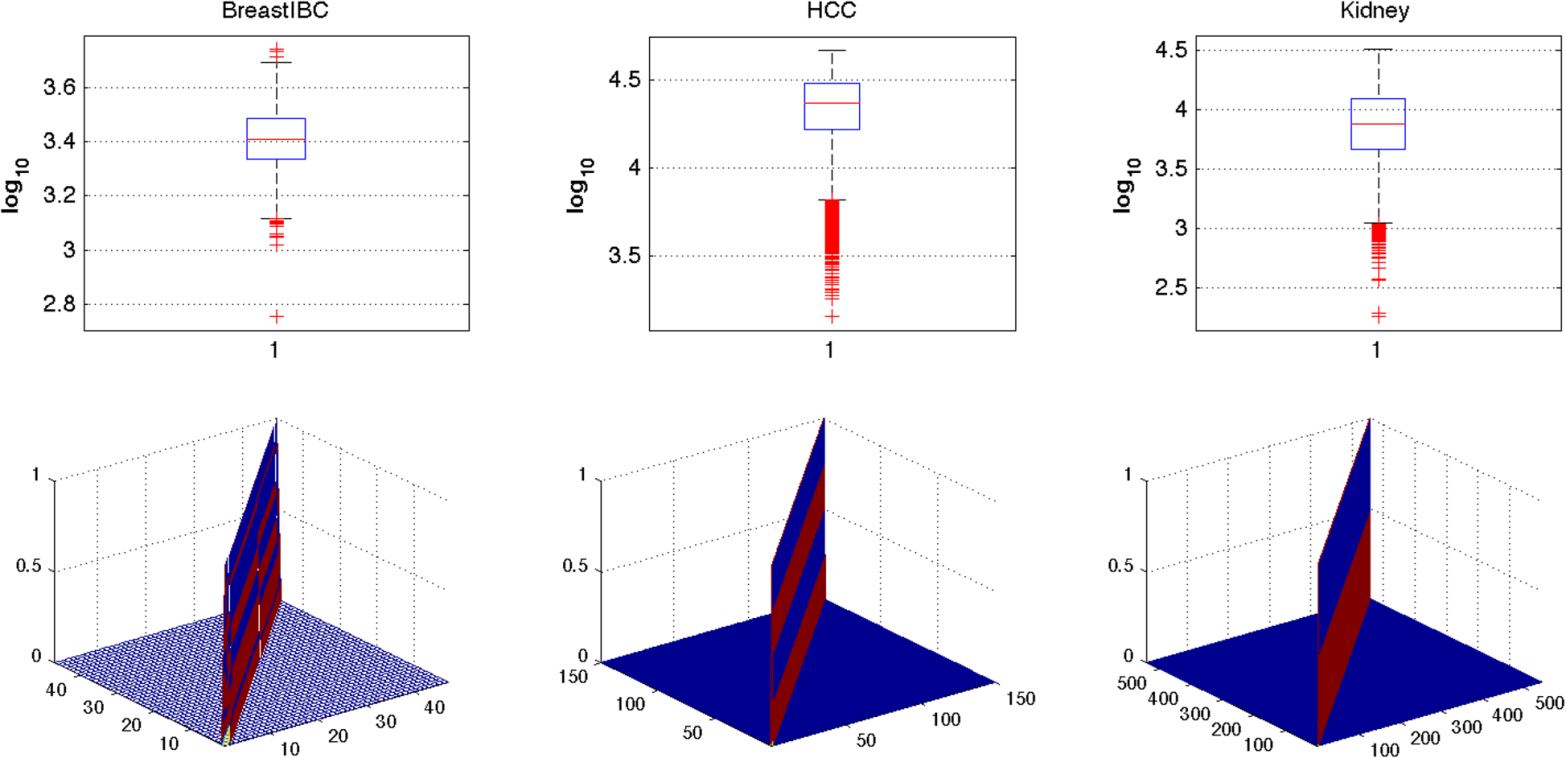
The kernel matrices of the overfitting bias. The first row illustrates the box-plots of all pairwise sample distance squares in each data. The second row lists the kernel matrices of the three data sets under the *‘rbf’* kernel (*σ*=1), where each data is viewed as the population of training data, are identity matrices

Figure 1 illustrates the box-plots of all pairwise sample distance squares $d_{\textit {ij}}^{2}, (i\neq j)$ in each data set in the first row of plots and kernel matrices of the three data sets under the *‘rbf’* kernel in the second row of plots by viewing each data set as the population of training data. It is interesting to see that the the minimum $d_{\textit {ij}}^{2}$ are greater than 10^2^, which means the distance between any two samples in the feature space will be approximately zero: *k*(*x*_*i*_,*x*_*j*_)≤ exp(−10^2^/2)∼10^−22^. As a result, the corresponding kernel matrix will be an identity matrix as illustrated by the corresponding plot in the second row.

It is noted that the large or even huge pairwise sample distances in each omics dataset are actually rooted in the molecular signal amplification mechanism in high-throughput profiling, where gene array, protein array and RNA-Seq technologies all employ real-time PCR or similar approaches to amplify gene and protein expression levels exponentially [14, 15]. As a result, the molecular signals greatly increase the sensitivity of disease phenotype and corresponding genotypes in diagnostics [28]. On the other hand, the pairwise distances between two samples are large or even huge mathematically, even if each sample is standardized as a zero-mean point with unit standard deviation.

**Fig. 2:**
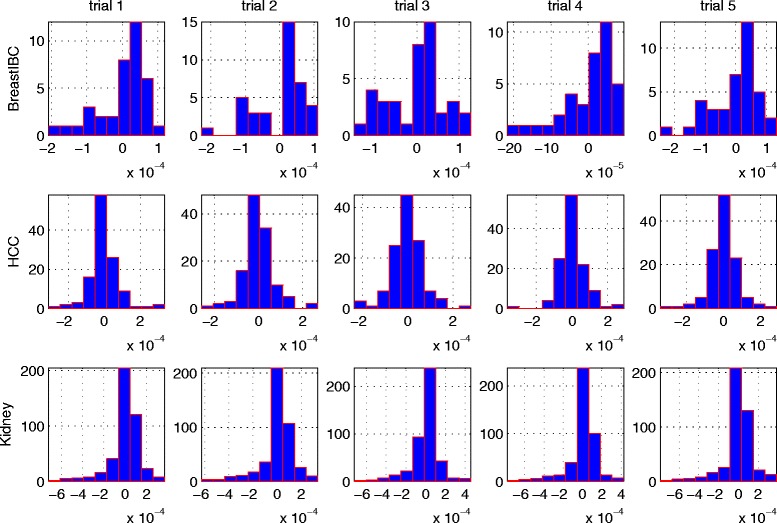
The distributions of *α* values. The distributions of *α* values of each diagnostic trial in the 5-fold cross validation for three data sets. The skewness of sample label distribution leads to the skewness of the distributions of *α* values of the diagnoses of the *BreastIBC* and *Kidney* data sets. The signs of the *α* values indicate the group property of corresponding support vectors. As such, more support vectors can be found for the majority-count type, which will increase the likelihood of an unknown sample to be detected as the majority-count type in diagnosis

The label skewness bias is due to the skewness of the label distributions that lead to there are more support vectors from the majority-count type samples and the class type of an unknown sample is more likely to be determined as the majority-count type. Figure 2 shows the distributions of *α* values, i.e., the Lagrange multipliers’ values: *α*_1_,*α*_2_⋯*α*_*m*_ in the dual problem, in each diagnostic trial in the 5-fold cross validation. As the weights of corresponding support vectors, its values are always positive or zero as we pointed out before. However, the sign of a weight is assigned in our SVM implementation for the convenience of indicating its class property, i.e. a positive (negative) sign means this weight (e.g. *α*_1_) is for the support vector belonging to the positive (negative) target group. It is easy to detect that the distributions of *α* values are nearly balanced for the *Hepatocellular carcinoma (HCC)* data that has a relatively balanced sample label distributions, where the number of positive signs are almost equal as that of the negative signs. However, the the distributions of *α* values of the *BreastIBC* and *Kidney* data are obviously skewed to the positive targets, which are the majority-count samples in each data set. In other words, more support vectors can be found for the majority-count type, which will increase the likelihood of an unknown sample to be detected as the majority-count type in the following decision making. For example, since there are 256 and 178 *α* values carrying the positive and negative signs respectively in the 5^*th*^ trial of diagnosis for the *Kidney* data, there will be a more likelihood for a test sample to be detected as a positive target.

On the other hand, the corresponding *b* values, which are the intercepts of the hyperplane that separates the two groups in the normalized data space, are all positive in each trial. For example, the *b* values of the five diagnostic trials for the *Kidney*and *BreastIBC* data are [0.7425, 0.7603, 0.7333, 0.7649, 0.7465] and [0.4594, 0.4210, 0.4594, 0.4594, 0.4359] respectively. As such, given a test sample *x*^′^, the decision function $f(x')=sign((\sum _{i=1}^{k}\alpha _{i}k(x',x_{i})+b)$ is more likely to determine it as the positive type, because most support vectors are from the positive type (the majority-count type) and the intercept value *b* is positive.

The underfitting bias is caused by the inappropriate kernel function such as *‘mlp’* that results in a kernel matrix with all entries are ‘1’s that has no any capability to distinguish different samples. To some degree, it corresponds an extreme case for an SVM classifier under the Gaussian kernel with a too large bandwidth parameter that also leads to the kernel matrix with all ‘1’ entries. It is noted that the underfitting bias is also independent of input data label distributions as the overfitting and label-skewness bias, though it corresponds to a kernel matrix with all ‘1’ entries instead of an identity kernel matrix as the former or a normal kernel matrix as the latter.

**Fig. 3:**
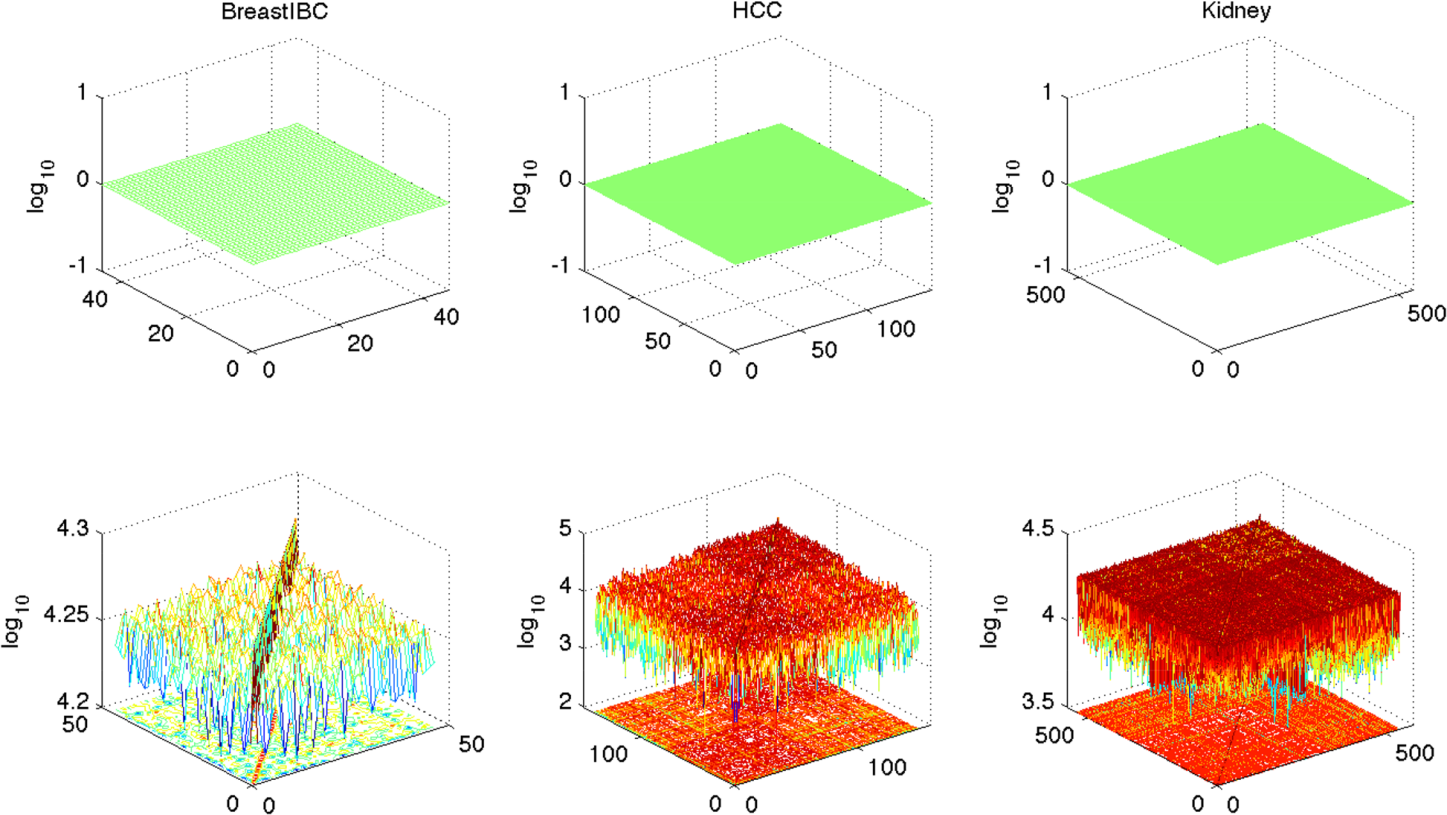
The comparisons of the kernel matrices in the label skewness and underfitting biases. The comparisons of the kernel matrices of the underfitting bias (*‘mlp’* kernels) and those of the linear kernels for the three data sets. The linear kernel matrices appear to be normal ones though the label skewness bias happens to the *BreastIBC* and *Kidney* data

Figure 3 shows the *‘mlp’* and *‘linear’* kernel matrices of the three data sets, where each data is treated as a training population. It is clear to see that the kernel matrices under the underfitting bias are flat matrices with all ‘1’ entries, but the kernel matrices under the linear kernel appear to be normal for all three data sets, even if there are *explicit* and *implicit* label skewness biases for the *BreastIBC* and *Kidney* data respectively.

#### Diagnostic bias conquering

There are no systematic approaches available to conquer diagnostic biases due to the gap between machine learning and translational bioinformatics [10]. Although previously related work has been proposed to investigate imbalanced data in SVM classification in data mining, all of these work mainly focus on the *‘imbalanced data’* where the sample label distributions are extremely imbalanced (e.g., 99.5 % positive labels and 0.5 % negative labels) [29, 30]. Moreover, these imbalanced data are not high-through omics data that do not have *‘large number of variables but small number of observations’* characteristics shared by all high-throughput omics data [11]. Thus, a more general but omics data focused algorithm is needed to overcome the diagnostic biases.

The overfitting and underfitting biases can be ‘conquered’ by avoiding using the corresponding kernels that lead to the identity, nearly identity, or all ‘1’ entries kernel matrices. However, it can be challenging to conquer the label skewness bias, especially the implicit diagnostic bias case that has *‘reasonable’* diagnostic accuracy but unbalanced sensitivity and specificity.

In this work, we propose a derivative component analysis (DCA) based support vector machines (DCA-SVM) to conquer the label skewness bias by extracting true signals by digging latent data characteristics from an input data [16]. The true signals share the same dimensionally with the original data but capture essential data characteristics. We introduce DCA briefly as follows and more details about this algorithm can be found in Han’s previous work on DCA [16].

### Derivative component analysis (DCA)

1. **Input:***X*^*t*^=[*x*_1_,*x*_2_⋯*x*_*n*_],*x*_*i*_∈ℜ^*p*^, DWT level *J*; cutoff *τ*; wavelet *ψ*, variability explanation threshold *ρ*
2. **Output:** true signals: *X*^∗^
3. **Step 1:** Conduct*J*-level DWT with wavelet *ψ* for *X*^*t*^ to obtain coefficient detail *cD*_*j*_ and approximation matrix *cA*:[*cD*_1_,*cD*_2_,⋯,*cD*_*J*_;*cA*_*J*_], where ${cD}_{j}\in \Re ^{p_{j}\times n}, {cA}_{J}\in \Re ^{p_{J}\times n},p_{j}=\left \lceil {{p}/{2^{j}}}\right \rceil $.
4. **Step 2:** Extract subtle data characteristics, remove system noise and retrieve global data characteristics
  - Conduct PCA for *cD*_*j*_,1≤*j*≤*τ* to obtain its PC matrix *U* and score matrix *S*: $U=[u_{1},u_{2},\cdots u_{p_{j}}], u_{i}\in \Re ^{n}$ and score matrix $S=[s_{1},s_{2}\cdots s_{p_{j}}], s_{i}\in \Re ^{p_{j}}, i=1,2\cdots p_{j}$.
  - Identify PCs *u*_*i*_,*u*_2_⋯*u*_*m*_, such that its variability explanation ratio *ρ*_*m*_≥*ρ*
  - Reconstruct ${cD}_{j}\leftarrow \frac {1}{p_{j}}{cD}_{j}\vec {(1)}\vec {(1)}^{T}+\sum _{i=1}^{m}u_{i}\times {s_{i}^{T}}, \vec {(1)}\in \Re ^{p_{j}}$ with all entries being ‘1’s
  - Reconstruct *cD*_*j*_,*τ*≤*j*≤*J* and *cA*_*J*_ under the variability explanation ratio at least 95 %
5. **Step 3:** Approximate the original data by the corresponding inverse DWT with the wavelet *X*^∗^←*inverseDWT*([*cD*_1_,*cD*_2_⋯*cD*_*J*_;*cA*_*J*_]).

In our implementation, we uniformly set the transform level *J*=7 for the wavelet *`db*8^′^, cutoff *τ*=2, and apply the first PC-based detail coefficient matrix reconstruction in DCA for the convenience of implementations [16, 31].

### Derivative component analysis based support vector machines (DCA-SVM)

Given training data *X*=[*x*_1_,*x*_2_⋯*x*_*p*_]^*T*^ and their labels $\{x_{i},c_{i}\}_{i=1,}^{p}c_{i}\!\in \!\{-1,1\},$ its corresponding true signals *Y*=[*y*_1_,*y*_2_⋯*y*_*p*_]^*T*^ are computed by using DCA, Then, a maximum-margin hyperplane: *O*_*h*_:*w*^*T*^*ϕ*(*y*)+*b*=0 in the feature space is constructed to separate the ‘+1’ (‘cancer’) and ‘-1’ (‘control’) types of the samples in true signals *Y*, which is equivalent to solving the following optimization problem with a parameter *μ*>0,

(2) $$ \begin{array}{c} \text{\ensuremath{\text{min}_{w,b,e}}}\frac{1}{2}w^{T}w+\frac{1}{2\mu}\sum_{i=1}^{p}\left(c_{i}-w^{T}\phi(y_{i})-b\right)^{2}\\ \text{s.t.}\,\ensuremath{e_{i}=c_{i}-w^{T}\phi(y_{i})-b},i=1,2\cdots p\\ \end{array}  $$

The dual problem of this constrained minimization problem can be formulated as follows, where *k*(*y*_*i*_,*y*_*j*_)=(*ϕ*(*y*_*i*_)·*ϕ*(*y*_*j*_))

(3) $$ \begin{array}{c} \sum_{i=1}^{p}\alpha_{i}k(y_{i},y_{j})+b+\mu=c_{i},\text{}\text{}i=1,2\cdots p\\ s.t. \sum_{i=1}^{p}\alpha_{i}=0\\ \end{array}  $$

The *b* and *α*_*i*_,*i*=1,2⋯*p* can be obtained by solving the corresponding linear system of the dual problem. The decision rule $f(x')=sign\left (\sum _{i=1}^{p}\alpha _{i}k(y_{i},y')+b\right)$ is used to determine the class type of a testing sample *x*^′^, where *y*^′^ is its corresponding vector computed from DCA. The function *k*(*y*_*i*_,*y*^′^) is a kernel function mapping *y*_*i*_ and *y*^′^ into a same-dimensional or high-dimensional feature space, which is chosen as the linear kernel *k*(*y*_*i*_,*y*^′^)=(*y*_*i*_·*y*^′^) in our experiment.

### Random undersampling Boost (*RUBoost*)

To demonstrate the effectiveness of the proposed algorithm, we include an ensemble learning method: random undersampling Boost (*RUBoost*) as well as the original SVM as comparison algorithms [29]. The reason we choose the ensemble learning method is because it is believed to perform well for imbalanced data [29, 30, 32]. We employ an ensemble of 1000 deep trees that have minimal leaf size of 5 with a learning rate 0.1 in *RUBoost* learning to attain a high ensemble accuracy.

Table 3 compares the performance of the proposed DCA-SVM with those of SVM and *RUBoost* under the 5-fold cross validation. It is interesting to see that our algorithm not only fully conquer the label skewness biases for the *BreastIBC* and *Kidney* data, but also achieve exceptional diagnostic results for all three data sets for its latent data characteristics extraction that forces a data characteristics driven diagnosis. It is noted that the extracted latent data characteristics contribute to the structure optimization of the kernel matrices that enhance the classifier’s detectability [31, 33, 34].

**Table 3:** The three diagnostics under 5-fold cross validation

Algorithm	Accuracy ± std (%)	Sensitivity ± std (%)	Specificity ± std (%)	NPR ± std (%)	PPR ± std (%)
		*BreastIBC data*			
*DCA-SVM*	97.78 ± 04.97	100.0 ± 00.00	90.00 ± 22.36	100.0 ± 00.00	97.50 ± 05.59
*SVM-linear*	74.56 ± 04.52	97.14 ± 06.39	16.67 ± 23.67	NaN	75.70 ± 06.52
*RUBoost*	73.33 ± 00.00	53.33 ± 44.72	82.86 ± 18.63	83.17 ± 15.86	54.67 ± 44.07
		*HCC data*			
*DCA-SVM*	99.33 ± 01.49	100.0 ± 00.00	98.57 ± 03.19	100.0 ± 00.00	98.82 ± 02.63
*SVM-linear*	94.02 ± 01.43	95.81 ± 03.83	92.42 ± 05.21	96.17 ± 03.50	92.39 ± 05.00
*RUBoost*	85.23 ± 00.00	82.08 ± 11.98	88.76 ± 06.30	82.56 ± 10.14	88.64 ± 06.54
		*Kidney data*			
*DCA-SVM*	99.81 ± 00.41	99.79 ± 00.47	100.0 ± 00.00	98.57 ± 03.19	100.0 ± 00.00
*SVM-linear*	90.23 ± 02.35	96.84 ± 03.07	44.07 ± 06.63	71.46 ± 16.90	92.38 ± 00.71
*RUBoost*	87.47 ± 00.00	90.95 ± 03.54	63.08 ± 11.17	51.31 ± 12.17	94.55 ± 01.42

For example, the explicit label skewness diagnostic bias illustrated in the *BreastIBC* data is overcome by achieving 97.78 % diagnostic accuracy with 100 % sensitivity and 90 % specificity. Unlike all negative targets are recognized as the positive targets in some diagnostic trial, the total negative prediction rate (NPR) is 100 % and the positive prediction rate (PPR) is 97 %. Moreover, the implicit label skewness diagnostic bias illustrated in the *Kidney* data is overcome by achieving 99.81 % diagnostic accuracy with 99.79 % sensitivity and 100 % specificity, compared to the original 90.23 % diagnostic accuracy with 96.84 % sensitivity and 44.07 % specificity.

Furthermore, DCA-SVM achieves the exceptional diagnostics on the *HCC*data by attaining 99.33 % diagnostic accuracy with 100 % sensitivity and 98.57 % specificity compared to the original 94.02 % accuracy with 95.81 % sensitivity and 92.42 % specificity. Alternatively, the *RUBoost* diagnosis has some improvements in balancing the sensitivity and specificity, whereas it has relatively low diagnostic accuracy, especially for balanced *HCC* data, and needs a long learning time.

**Fig. 4:**
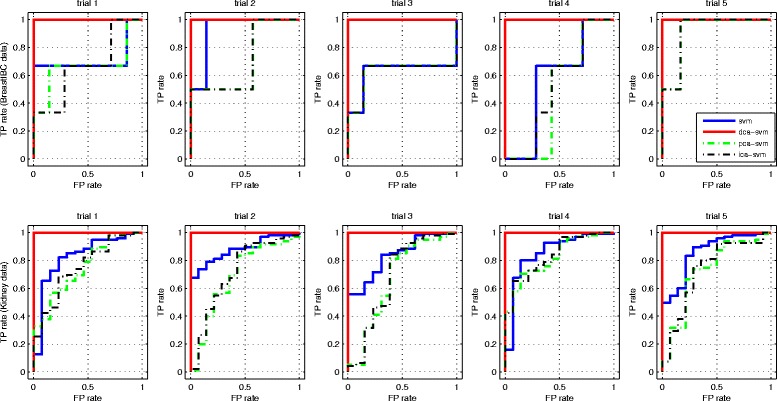
ROC plots. The ROC plots of DCA-SVM, SVM, PCA-SVM, ICA-SVM diagnoses under the 5-fold cross validation for the *BreastIBC* and *Kidney* data

**Fig. 5:**
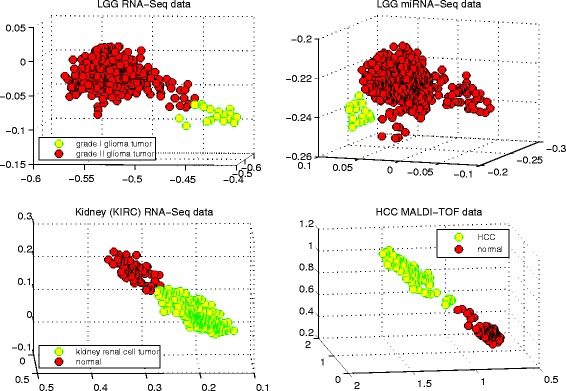
The phenotype separation. The phenotype separation for four different data sets: *GliomaRNASeq* (LGG RNA-Seq), *GliomaMiRNASeq* (LGG MiRNA-Seq), *Kidney* (Kidney (KIRC) RNA-Seq), and *HCC* (HCC MALDI-TOF) by using the top three biomarkers

Figure 4 compares the ROC plots of DCA-SVM, SVM, PCA-SVM, ICA-SVM diagnoses under the 5-fold cross validation for the *BreastIBC* and *Kidney* data [16, 33]. It is easy to see that the proposed DCA-SVM diagnosis conquers the label skewness bias by achieving the best performance, which prepares itself as a good candidate in personalized diagnostics in the coming personalized medicine for its unbiased exceptional diagnostic performance for different omics data. It is worthwhile to point out that such a rivaling clinical-level diagnosis is mainly because the true signals extraction in DCA that forces the SVM hyperplane construction to rely on both subtle and global data characteristics of the whole profile in a de-noised feature space, which seems to contribute to a robust and consistent high-accuracy diagnosis greatly. In fact, since such a consistent performance applies to different data sets rather than work only on an individual data set, it almost prevents from any overfitting possibility. Moreover, the following two subsections further demonstrate such an exceptional performance is impossible from overfitting because our proposed algorithm works well consistently for different data sets with different training and test data selection methods. Especially, the phenotype separation results in Fig. 5 strongly validate the effectiveness from a biomarker discovery and visualization standing point.

#### Independent data sets: brain low grade glioma (LGG) TCGA data

To further demonstrate the effectiveness of our proposed algorithm, we have retrieved level-3 TCGA data for brain low grade gliomas (LGG) from the TCGA portal that include gene expression, protein expression, RNA-Seq and miRNA-Seq data [22, 35]. The LGG refers to the grade I and grade II glioma tumors that are usually considered as benign brain tumors compared with those grade II and IV glioma tumors. Since the gene and protein expression data only contain grade-I glioma samples that prevent us doing diagnostics from a translational bioinformatics viewpoint, we include the RNA-Seq and miRNA-Seq data as the independent data sets: *GliomaRNASeq* and *GliomaMiRNASeq* for our algorithm testing. The detailed information about the two data sets can be found in the Table 4, where each feature refers to a gene or microRNA.

**Table 4:** Brain Low grade Glioma (LGG) TCGA data

Data	#Feature	#Sample	Technology	Platform
*GliomaRNASeq*	20,531	18 *grade-I Glioma tumors* +		
		*516 grade-II Glioma tumors*	RNA-Seq	IlluminaHiSeq_RNASeqV2
*GliomaMiRNASeq*	1046	18 *grade-I Glioma tumors* +		
		*512 grade-II Glioma tumors*	miRNA-Seq	IlluminaHiSeq_miRNASeq

#### Normalization

It is noted that both are *‘imbalanced data’*, where 96.63 % and 95.88 % samples are grade-II tumors respectively, and follow the negative binomial (NB) distribution approximately. The raw *GliomaRNASeq* data, a big data that asks 14.5 Gigebytes storage, is normalized by dividing each sample with a scale factor *s*=*Q*_3_/1000, where *Q*_3_ is the 75-percentile of each sample. The raw data is normalized by the *count-per-million* method, in which all counts in a sample are adjusted to reads per million to facilitate comparison between samples [36].

#### Monte Carlo simulation oriented training and test data selection

Different from the previous *k*-fold cross-validation, we randomly select 50 % of Glioma RNA-Seq (miRNA-Seq) samples for training and another 50 % for test, and repeat such a process 500 times in our diagnostic experiments. It is noted that such a Monte Carlo simulation oriented independent training and test data choice will have an advantage to evaluate the effectiveness of the proposed algorithm than the previous *k*-fold cross-validation. This is because it reduces the dependence between training and test data by fully leveraging the two omics data sets with a large number of observations.

Table 5 compares the diagnostic results of DCA-SVM, with SVM under four different kernels: *‘linear’*, *‘rbf’*, *‘quad’* and *‘mlp’*for the two data sets. It is not a surprise that the SVM-*mlp*classifier encounters the underfitting bias for both *LGG* data sets by demonstrating quite low diagnostic accuracy values. Similarly, the SVM-*rbf* classifier still suffers from the overfitting bias by only recognizing the majority count phenotypes. That is, its average diagnostic accuracy closely approximates the majority count ratios of the *GliomaRNASeq* and *GliomaMiRNASeq* data sets $96.68\,\%\approx \frac {516}{516+18}$ and $96.63\,\%\approx \frac {512}{512+18}$ respectively. For the same reason, its average positive prediction rate will just be its diagnostic accuracy because the SVM-*rbf* classifier diagnoses all samples into the positive samples. Alternatively, the corresponding negative prediction ratio $NPR=\frac {TN}{TN+FN}$ is NaN because of *TN*=*FN*=0 in each diagnostic case, and the sensitivity and specificity are 100 % and 0 % respectively.

**Table 5:** The diagnostic results with independent training and test sets for LGG data

Algorithm	Accuracy ± std (%)	Sensitivity ± std (%)	Specificity ± std (%)	NPR ± std (%)	PPR ± std (%)
		*GliomaRNASeq*			
*DCA-SVM*	99.52 ± 00.58	99.64 ± 00.53	97.00 ± 08.08	91.18 ± 11.05	99.87 ± 00.36
*SVM-linear*	95.87 ± 00.84	98.77 ± 00.00	12.10 ± 11.46	NaN	97.02 ± 00.85
*SVM-rbf*	96.68 ± 00.78	100.0 ± 00.00	00.00 ± 00.00	NaN	96.68 ± 00.78
*SVM-quad*	96.53 ± 00.75	99.60 ± 00.40	07.40 ± 08.67	NaN	96.91 ± 00.79
*SVM-mlp*	56.28 ± 05.61	56.73 ± 05.95	43.77 ± 18.61	03.34 ± 01.53	96.70 ± 01.31
		*GliomaMiRNASeq*			
*DCA-SVM*	99.63 ± 00.52	99.73 ± 00.39	97.52 ± 08.21	93.13 ± 09.40	99.89 ± 00.39
*SVM-linear*	93.78 ± 01.27	96.68 ± 01.58	10.93 ± 10.04	10.89 ± 11.08	96.89 ± 00.84
*SVM-rbf*	96.63 ± 00.81	100.0 ± 00.00	00.00 ± 00.00	NaN	96.63 ± 00.81
*SVM-quad*	95.65 ± 00.97	98.76 ± 00.87	06.14 ± 07.39	NaN	96.80 ± 00.79
*SVM-mlp*	56.62 ± 06.31	58.16 ± 06.69	42.51 ± 18.98	03.45 ± 01.74	96.68 ± 01.30

Also like the previous cases, the SVM-*linear* and SVM-*quad* classifiers both encounter the explicit label skewness bias because both data sets are imbalanced where the *GliomaRNASeq* data has 18 grade I and 516 grade II gliomas and the *GliomaMiRNASeq*data has 18 grade I and 512 grade II gliomas respectively.

The explicit label skewness bias demonstrates a deceptive diagnostic accuracy that is close to the majority-count ratio for each data. For example, the SVM-*linear* classifier achieves an average accuracy 95.87 % and 93.78 % for the two data sets respectively, both of which are close to the majority-count ratios 96.68 *%* and 96.63 *%*. However, both diagnostic results are characterized by imbalanced sensitivity & specificity, and positive & negative prediction rates. For example, the SVM-*linear* classifier achieves 98.77 % sensitivity and 12.10 % specificity.

Although its average negative predication ratio (NPR) appears to be NaN, such an exception is caused by the fact that both *TN* and *FN* are zero counts in some trials of diagnosis, due to the major-count phenotype favor mechanism. In fact, it is easy to estimate that its average NPR should be a small percentage, because the corresponding average PPR is 97.02 *%*, i.e. very few negative targets or even none are correctly diagnosed in each diagnosis. As such, the *‘high’* diagnostic accuracy does not mean the classifiers have high detection capabilities. Instead, the high’ diagnostic accuracy is from the high majority-count ratio.

However, the proposed DCA-SVM algorithm successfully overcomes the diagnostic biases and achieves rivaling-clinical diagnostic accuracy and balanced sensitivity and specificity for the two data sets. In particular, we still employ the transform level *J*=7 and cutoff *τ*=2, in addition to keeping the first PC-based detail coefficient matrix reconstruction in DCA for the sake of consistence.

Such a result is consistent with the previous results from gene/protein expression and RNA-Seq data with *k*-fold cross validation. For example, our DCA-SVM classifier achieves 99.52 % (sensitivity: 99.64 %, specificity: 97.00 %, NPR: 91.98 %, PPR: 99.87 %) and 99.63 % (sensitivity: 99.73 %, specificity: 97.52 %, NPR: 93.13 %, PPR: 99.89 %) average diagnostic accuracy for the *GliomaRNASeq*and *GliomaMiRNASeq*data. Considering different types of omics data and different training and test data selections, such a result strongly suggests the effectiveness of our proposed method in conquering the diagnostic biases.

#### Diagnostic index

We create a diagnostic index $\beta =-\log _{2}a-\log _{2}\frac {s+p}{2},$ where *a*,*s*, and *p* represent accuracy, sensitivity and specificity to evaluate if a classifier is subject to any diagnostic biases. A small diagnostic index value (e.g., *β*=0.01) means the classifier achieves a good accuracy with a light degree diagnostic bias. The smallest diagnostic index refers to the perfect diagnosis for a classifier: *a*=*s*=*p*=100 *%*. Alternatively, a large *β* (e.g., 2.0) means classifier achieves a poor diagnostic accuracy or a high degree diagnostic bias. Table 6 compares the diagnostic index values of the proposed DCA-SVM with those of the other classifiers. It is interesting to see that its *β* values are the lowest among all diagnostic index values, which validate again the effectiveness of the proposed algorithm in conquering the label skewness bias and achieving rivaling clinical diagnostic results.

**Table 6:** The diagnostic index

	Diagnostic index	
Algorithm	*GliomaRNASeq*	*GliomaMiRNASeq*
*DCA-SVM*	0.0314	0.0235
*SVM-linear*	0.9123	0.9868
*SVM-rbf*	1.0487	1.0495
*SVM-quad*	0.9533	0.9951
*SVM-mlp*	1.8221	1.7857

#### Derivative component analysis based phenotype separation

We create a diagnostic index $\beta =-\log _{2}a-\log _{2}\frac {s+p}{2}$, where *a*,*s*, and *p* represent accuracy, sensitivity and specificity to evaluate if a classifier is subject to any diagnostic biases. A small diagnostic index value (e.g., *β*=0.01) means the classifier achieves a good accuracy with a light degree diagnostic bias. The smallest diagnostic index refers to the perfect diagnosis for a classifier: *a*=*s*=*p*=100 *%*. Alternatively, a large *β* (e.g., 2.0) means classifier achieves a poor diagnostic accuracy or a high degree diagnostic bias. Table 6 compares the diagnostic index values of the proposed DCA-SVM with those of the other classifiers. It is interesting to see that its *β* values are the lowest among all diagnostic index values, which validate again the effectiveness of the proposed algorithm in conquering the label skewness bias and achieving rivaling clinical diagnostic results.

#### Derivative component analysis based phenotype separation

The diagnostic results from the proposed DCA-SVM classifier indicates that the high-dimensional omics data in our experiment are linear separable after derivative component analysis. In other words, it means that support vectors can be found to separate the two groups of samples geometrically according to the definition of linear separability [12]. On the other hand, it suggests that disease biomarkers can be identified from the omics data to discriminate different phenotypes in such a translational bioinformatics based disease diagnostics. As such, we demonstrate the following biomarker discovery method that captures disease biomarkers and a visualization technique that show the possible support vectors in phenotype separation, that is to further ‘prove’ and validate the effectiveness of our proposed algorithm.

Our biomarker discovery method assumes the normal distribution of input data. If an input data is not normally distributed, we conduct a transform **Y=E(log(X+1))/var(log(X+1))** to covert it to a corresponding normally distributed data approximately. It is noted that **log(X+1)** is obtained by element-wisely applying the log transform to **X+1,** which adds each entry in input data **X** by 1. Similarly, **E(log(X+1))** updates **log(X+1)** by adjusting its column with its corresponding mean, and **var(log(X+1))** is the matrix, each column of which is a vector consisting of the variance of **log(X+1)** at the column, and Y is obtained by the element-wise division between **E(log(X+1))** and **var(log(X+1))**.

Then, derivative component analysis (DCA) is applied to the normally distributed omics data to retrieve its true signals by using the same parameter setting in the previous experiments. Finally, the classic two-sample *t-test* is employed to identify the differentially expressed features (e.g. genes) with the smallest *p*-values from the extracted true signals as potential biomarkers. It is worthwhile to point out that a large amount of tiny *p*-values will come from the *t*-test due to the de-noising process in DCA. Although we can get a set of well-supported biomarkers from the statistical test applied to the true signals, we prefer to employ the top three biomarkers to conduct phenotype separation and corresponding support vector finding for the convenience of visualization.

Figure 5 shows the corresponding phenotype separations for four data sets from different high-throughput technologies and platforms: *GliomaRNASeq* (LGG RNA-Seq), *GliomaMiRNASeq* (LGG MiRNA-Seq), *Kidney* (Kidney (KIRC) RNA-Seq), and *HCC* (HCC MALDI-TOF), by using its top three biomarkers. Each yellow/red dot in the visualization represents a corresponding sample. For example, the 18 yellow dots represent 18 grade I glioma samples in the NW plot for LGG RNA-Seq data. It is interesting to see that the three biomarkers discovered from each data set demonstrate the linear-separability very well and corresponding support vectors can be easily found from each phenotype separation.

Such results strongly suggest the effectiveness of our proposed algorithm and provides a visualization support for DCA-SVM’s rivaling clinical diagnostic performance. Furthermore, it provides more insights to elucidate the latent structures of the omics data, which can contribute to deciphering the different pathological sub-states of tumors. For example, the NE sub-figure discloses that 512 grade II tumors of the *GliomaMiRNASeq* data span three different clusters, which may indicate that grade II tumors may have different pathological sub-states due to different genetic alternations [35]. It is also noted that such results also apply to the *BreastIBC data* though it is not included in Fig. 5.

## Discussion

In this work, we comprehensively investigate diagnostic bias in translational bioinformatics by using support vector machines (SVM). It is worthwhile to point that the overfitting bias and underfitting bias can be viewed as special diagnostic biases associated with the kernel-based learning, though they still happen in the other classifier-based diagnosis. However, the label skewness bias can be found widely found in the other classifiers, because the SVM classifiers with different kernels can be viewed as the ‘simulations’ of different classifiers [12]. For example, an *SVM-linear* classifier can be viewed as a simulation of linear discriminant analysis (LDA), because they usually have a similar or same level performance [37]. In fact, LDA does demonstrate label skewness diagnostic bias on the *BreastIBC* data under the same cross validation by achieving 71.83 % accuracy with 94.17 % sensitivity and 15 % specificity.

We also have employed a multi-layer perceptron (MLP) classifier to the five data sets used to investigate the occurrence of diagnostic biases for its comparable performance with respect to SVM and other classifiers such as decision trees [38, 39]. We still use the 5-fold cross validation is still for the convenience of comparisons. The MLP classifier has 10 neurons in its input layer, two hidden layers, each of which has 5 neurons, and two neurons in its output layer. The Levenberg-Marquardt optimization is employed to train the network, in which the maximum number of epochs and minimum performance gradient in training are set as 10^3^ and 10^−9^ respectively [40]. We are interesting to find that it encounters different diagnostic biases on almost all data sets under the 5-fold cross validation except the *Hepatocellular carcinoma (HCC)* data, where it has an accuracy 85.91 % with sensitivity 90.29 % and specificity 81.92 %. For example, it achieves 92.18 % accuracy (sensitivity 95.40 %, specificity: 0.0 %) for the *GliomaRNASeq*data, and 96.07 % accuracy (sensitivity 99.40 %, specificity: 1.08 %) for the *GliomaMiRNASeq* data respectively. Obviously, it encounters overfitting diagnosis by diagnosing all test samples as the majority count samples with an approximately zero specificity. In addition, it demonstrates the explicit label skewness biases for the *Kidney* and *BreastIBC* data with low diagnostic accuracy: 79.73 % (sensitivity: 14.45 %, specificity: 89.09 %) and 65.78 % (sensitivity: 85.71 %, specificity: 13.33 %) respectively. All these results strongly demonstrate the generalization of our proposed diagnostic biases.

Unlike other ad-hoc diagnostic bias conquering by tuning parameters, the proposed DCA-SVM demonstrates rivaling-clinical level diagnostic results by overcoming both explicit and implicit label skewness biases. Although some statistical test-based feature selection can conquer some diagnostic bias well for some data, it may not be generalized to other data with different distributions. For example, the *SVM-linear* classifier can achieve a quite excellent diagnostic performance on the *BreastIBC* data with an average diagnostic accuracy 98.00 % (sensitivity: 100 %, specificity: 93.33 %) under the *5*-fold cross validation, if we only pick the top-ranked 200 genes (features) from this data by using Bayesian *t-*test [41]. However, if we apply the same feature selection approach to the *Hepatocellular carcinoma (HCC)* data, the classifier only attains a mediocre performance with an average diagnostic accuracy 88.03 % (sensitivity: 84.76 %, specificity: 91.08 %), which is far from the more than 94 %-level diagnostic accuracy achieved by the same classifier without using any feature selection. On the other hand, such a normal distribution assumed feature selection method can not apply to the RNA-Seq and MiRNA-Seq data directly, because these data are not normally distributed. Thus, such a feature filtering approach can not be a good choice for overcoming diagnostic biases. Alternatively, our derivative component analysis (DCA) is a generic feature extraction algorithm that does not have special data distribution requirements but retrieve true signals from each omics data by capturing essential data behaviors. As such, the proposed DCA-SVM diagnosis can be viewed as a generic solution for the diagnostic bias problem in translational bioinformatics.

Although we assume training and testing samples are picked from a normalized population in our context, our method can still work well provide the testing samples are not normalized or normalized with a different approach as the training ones. The renormalization process will be required but it can be different for different types of omics data. For example, the renormalization for microarray data is usually done by normalizing all the training and testing samples before retraining the classifier in diagnostics [42, 43]. This is mainly because microarray data generally has strong background-signals that make the comparisons of expression levels between genes within a single sample impossible [44, 45]. Due to its fundamentally different data generation mechanism as microarray data, RNA-Seq or MiRNA-Seq data can compare different genes’ expression levels within a single sample [44]. As such, the renormalization for such type of data can be done by only conducting normalization for each testing sample by using corresponding normalization methods (e.g. DESeq-normalization) before the proposed diagnosis [24, 46].

## Conclusions

Our studies comprehensively investigate the diagnostic bias problem in translational bioinformatics by analyzing benchmark gene array, protein array, RNA-Seq and miRNA-Seq data. We identify three types of diagnostic biases: overfitting bias, label skewness bias, and underfitting bias in SVM diagnosis, and disclose the reasons for its occurrence through rigorous analysis. As we pointed out before, the diagnostic biases, which happen at almost all kernels and data with different distributions, are actually caused by three major factors, that is, kernel selection, special signal amplification mechanism in the high throughput profiling, and training data label distribution.

Interestingly, the overfitting bias and label skewness bias both demonstrate a majority-count phenotype favor mechanism in diagnosis, which means that only majority-count samples can be recognized in diagnosis. However, the former is rooted in the molecular signal amplification mechanism in high-throughput profiling that leads to the large or even huge pairwise distances in the training data. The latter is caused by the unbalanced label distributions in the training data.

Unlike other diagnostic biases, the label skewness bias is hard to detect and conquer, especially the implicit label skewness bias that usually demonstrate quite normal or even some good diagnostic accuracy but with imbalanced sensitivity and specificity. Our studies propose a DCA-SVM that not only conquer the bias but also achieve rivaling clinical diagnostic results by leverage the powerful feature extraction capabilities of derivative component analysis. Our work is not only significant in translational bioinformatics by identifying and solving an important problem, but also has a positive impact on machine learning for adding new results to kernel-based learning for omics data.

In our further studies, we plan to investigate the label skewness bias for the multi-class diagnostics, which can be more complicate and applied in medical informatics than the current binary type diagnostics [47]. Moreover, we are interested in investigating diagnostic biases in deep learning methods for its importance in big omics data oriented diagnostics [48, 49], in addition to integrating different types of omics data sets to conduct differential expression analysis [50].

## Availability of supporting data

All data sets used in this paper are publicly available from https://sites.google.com/site/tbdiagnosticbiases/.
